# Biology, Bionomics and Molecular Biology of *Anopheles sinensis* Wiedemann 1828 (Diptera: Culicidae), Main Malaria Vector in China

**DOI:** 10.3389/fmicb.2017.01473

**Published:** 2017-08-09

**Authors:** Xinyu Feng, Shaosen Zhang, Fang Huang, Li Zhang, Jun Feng, Zhigui Xia, Hejun Zhou, Wei Hu, Shuisen Zhou

**Affiliations:** ^1^National Institute of Parasitic Diseases, Chinese Center for Disease Control and Prevention Shanghai, China; ^2^Key Laboratory of Parasite and Vector Biology, National Health and Family Planning Commission Shanghai, China; ^3^WHO Collaborating Center for Tropical Diseases Shanghai, China; ^4^National Center for International Research on Tropical Diseases Shanghai, China; ^5^Joint Research Laboratory of Genetics and Ecology on Parasites-Hosts Interaction, National Institute of Parasitic Diseases – Fudan University Shanghai, China; ^6^Université de Montpellier, IES – Institut d’Electronique et des Systèmes, UMR 5214, CNRS-UM Montpellier, France; ^7^Cirad, UMR 17, Intertryp, Campus International de Baillarguet Montpellier, France; ^8^Institut de Recherche pour le Développement (IRD France), LIPMC, UMR-MD3, Faculté de Pharmacie Montpellier, France; ^9^Department of Microbiology and Microbial Engineering, School of Life Sciences, Fudan University Shanghai, China

**Keywords:** *Anopheles sinensis*, biology, bionomics, gene, protein, molecule, China, vector

## Abstract

China has set a goal to eliminate all malaria in the country by 2020, but it is unclear if current understanding of malaria vectors and transmission is sufficient to achieve this objective. *Anopheles sinensis* is the most widespread malaria vector specie in China, which is also responsible for vivax malaria outbreak in central China. We reviewed literature from 1954 to 2016 on *An. sinensis* with emphasis on biology, bionomics, and molecular biology. A total of 538 references were relevant and included. *An. sienesis* occurs in 29 Chinese provinces. Temperature can affect most life-history parameters. Most *An. sinensis* are zoophilic, but sometimes they are facultatively anthropophilic. Sporozoite analysis demonstrated *An. sinensis* efficacy on *Plasmodium vivax* transmission. *An. sinensis* was not stringently refractory to *P. falciparum* under experimental conditions, however, sporozoite was not found in salivary glands of field collected *An. sinensis*. The literature on *An. sienesis* biology and bionomics was abundant, but molecular studies, such as gene functions and mechanisms, were limited. Only 12 molecules (genes, proteins or enzymes) have been studied. In addition, there were considerable untapped omics resources for potential vector control tools. Existing information on *An. sienesis* could serve as a baseline for advanced research on biology, bionomics and genetics relevant to vector control strategies.

## Introduction

Malaria was once epidemic in China and disease levels were high. However, a significant decline of malaria incidence has occurred with reported cases declining from > 9 million cases in the 1960s to only 3078 cases in 2014 (Tang, 2010; Wang, 2014; Li et al., 2015). In 2010, the Chinese Government launched Malaria Elimination Program with a goal to eliminate malaria in the entire country by 2020 (Feng et al., 2014).

Four Anopheline species, *Anopheles sinensis, Anopheles anthropophagus, Anopheles minimus*, and *Anopheles dirus*, are considered main vectors for malaria transmission in China. Among these, *An. sinensis* is the most widely distributed species (Zhu et al., 2013). It is the most important malaria vector in flatlands, especially in the paddy planting regions. *An. sinensis* is considered to be a competent vector for *Plasmodium vivax* malaria since it is the only major vector in central China where *P. vivax* is prevalent, locally transmitted, and where several malaria epidemics have occurred (Zhou et al., 2007). Besides malaria, *An. sinensis* can also transmit lymphatic filariasis (Reid, 1968), JEV and *Rickettsia felis* (Scherer et al., 1959; Zhang et al., 2014).

The distribution, habitat, feeding behavior, and host selection of *An. sinensis* in China has been extensively studied. Ma (1954) published the first biology study of *An. sinensis* and since then, there have been many reports on its biology or bionomics. This information has contributed to the success of malaria control programs. Larval reduction by drainage, and filling, and IRS have been the main malaria control measures. The primary intervention measures for malaria elimination in China continue to target the adult vector by IRS or LLINs (Liu and Liu, 2010).

Interactions between vector and parasite are important in malaria transmission dynamics. Identification of molecules involved in multifaceted developmental cycles of parasites within the vector and the related mechanisms accounting for survival and proliferation can provide attractive targets to interfere in the disease transmission (Sreenivasamurthy et al., 2013). However, many of the molecules and mechanisms in *An. sinensis* are still remain unknown. Understanding the underlying details of the vector-pathogen interaction would underpin the prevention and control of parasitic diseases.

Although there are many studies on *An. sinensis* distribution, bionomics and molecular study in China, the information was notably dispersed in the literature. So, current studies were systematically reviewed. The objective of the present study was to review the biology, bionomics and molecules of *An. sinensis* in China. This could provide insights for development of novel mosquito control strategies and increase the effectiveness of the vector control interventions in elimination campaign.

## Methods

An electronic search of peer-reviewed scientific and medical literature published between January 1954 and September 2016 in Chinese and English was conducted using PubMed (MEDLINE), CNKI (China National Knowledge Infrastructure), VIP (Chongqing VIP Database), and CSPD (China Science Periodical Database, Wan Fang) and Web of Science databases. Gray literature and programmatic documents were also searched using Google, Google Scholar and other search engines using the same search terms.

**FIGURE 1:**
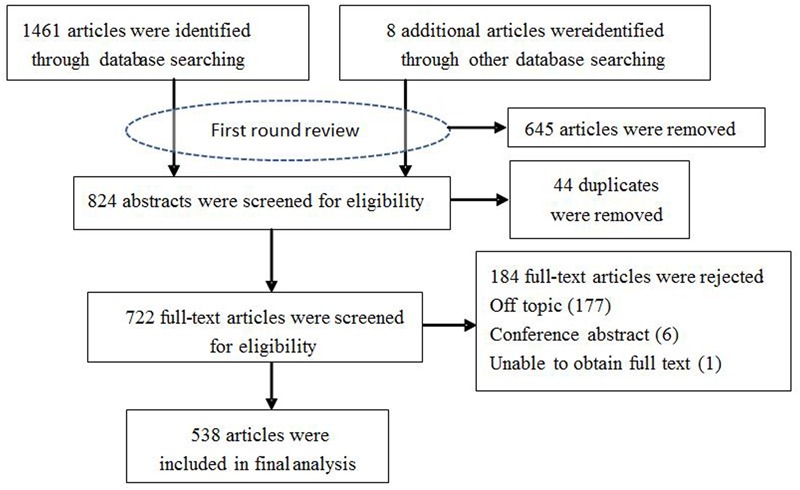
Flow diagram of the selection procedure used for the systematic review of accessible articles.

The following search terms (or their Chinese equivalents) were used: *Anopheles sinensis*, distribution, biology, bionomics, molecule, ecology and China. The decision tree for the inclusion or exclusion of articles is shown in **Figure 1**. Only publications reporting biology, bionomics, and molecules of *An. sinensis* from China were included. Articles submitting a report on morphology, development, reproductive, life cycle, vector competence, larval and adult ecology, vector capacity, molecules involved in physiology and pathology were included. Studies involving insecticides were excluded because it would be the focus of a future review. These data were extracted and processed through a series of rigorous checking procedures before classification into a database. All results were initially reviewed for mosquito bionomics (larval and adult ecology), biology and related molecular studies based on the title and abstract. Relevant publications were further reviewed using the full text to determine whether the data focused on distribution, bionomics, epidemiology, vector competence, or identified molecules.

## Results

**Figure 1** illustrates the search results. The initial search strategy generated 1469 records. After first round review of titles and abstracts, a total of 824 articles were amalgamated for review. After removing 44 duplicates, 722 relevant publications were further reviewed based on the full text to find out whether primary or secondary data on the biology, bionomics, molecules. Finally, a total of 538 papers and reports met these criteria. Selected articles were saved in Endnote and their characteristics corresponding to the criteria manually entered into Microsoft Excel for ongoing data management. From these articles, we assessed risk of bias for included studies but did not exclude studies on the basis. The outcomes of analysis by study area are outlined below.

## Biology

### Morphology

**FIGURE 2:**
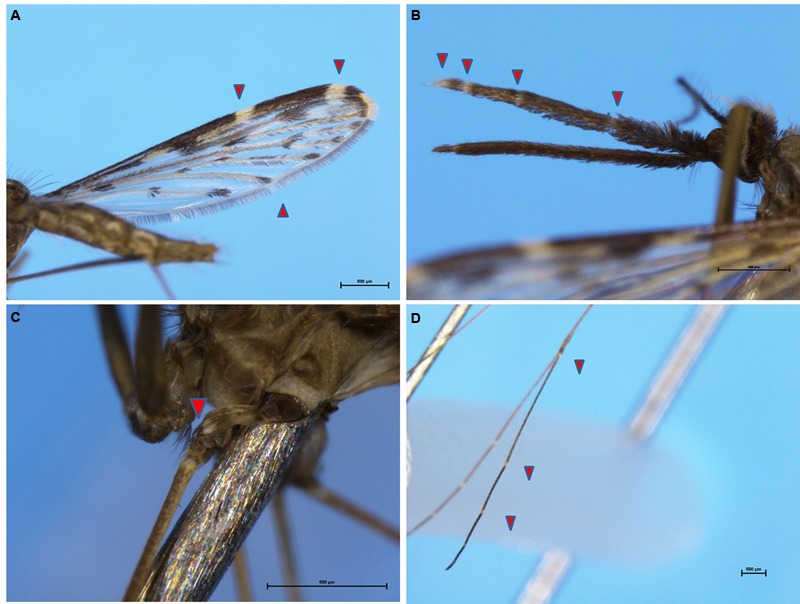
Typical morphological characteristics of *An. sinensis*. **(A)** A pale fringe spot at vein 5.2. **(B)** Four pale bands on the palpi. **(C)** Patch of pale scales at midcoxa. **(D)** Apical pale bands at hindtarsomeres.

*Anopheles sinensis* Wiedemann (1828) is a member of the *Anopheles* hyrcanus species group. Adults are morphologically distinguished from sibling species by the presence of four pale bands on the palpi, a fringe spot at vein 5.2, a tuft of dark scales on the clypeus on each side in the female (**Figure 2**) and a T-type speckle on ventral aspect (Feng and Zhang, 1962; Yang et al., 1994). Morphometric and morphological characteristics of *An. sinensis* eggs were studied using scanning electron microscopy (Zhang et al., 1982, 1984; Li D. et al., 2010). Eggs of *An. sinensis* were black (newly laid eggs were white and blackened about an hour later (Liu, 1986). They were boat-shaped in lateral, view with a mean length of 0.5mm. Floats present on sides of the egg surface and these had 7∼10 ribs.

### Development (Egg to Adult)

Like all mosquitoes, *An. sinensis* has four life stages: egg, larva, pupa, and adult. *An. sinensis* female lay individual egg on water surface. According to the studies, the number of eggs laid ranges from dozens to hundreds (Lu, 1984; Li et al., 1994; Qu et al., 2000i; Zhong, 2000; Zhong and Tan, 2000). The number of eggs laid were affected by the season (Qu et al., 2000i), temperature (Zhong, 2000; Zhong and Tan, 2000), blood resources (Su and Su, 1989), and experimental factors (Luo et al., 1988; Li and Tang, 2010). Luo et al. (1988) noted that the number of eggs laid in cow sheds was greater than in human dwellings and speculated this might be an adaption related to blood preference. Lu (1984) observed that most female laid eggs during entire night, and were prone to oviposit between dusk and dawn (usually from 7 pm to 5 am). The result was consistent with studies in other provinces of China (Liu, 1986; Xiang, 1988; Qu et al., 2000d,i). Generally the female adults began to lay eggs in around 3 days (2.57–3 days) after blood feeding, and its peaks started from 5 to 16 days (Fengwen et al., 1988; Qu et al., 2000a) in July and August (Qu et al., 2000d,i). Interestingly, in *An. gambiae s.s.*, Christiansenjucht et al. (2015) observed temperature had effect on the egg laying, the number of eggs laid was highest and lowest when adults were kept at 27°C and 31°C respectively. Differences were also observed among the successive blood meals in times to egg laying and hatching, number of eggs laid, and chances of feeding and egg laying.

Embryogenesis and hatching have been described in detail by different research teams (Wang et al., 1985; Li et al., 1994; Shen et al., 1995; Lai et al., 1995; Qu et al., 2000e). Generally it takes around 2.07–2.88 days (mean) or more for full embryonic development depending on the temperature and environment (Fengwen et al., 1988; Qu et al., 2000a; Zhong, 2000; Zhong and Tan, 2000). Hu et al. (1986) observed that the optimal temperature was between 25 and 28°C. At 25–30°C, *An. sinensis* hatched at 2.9 d after oviposition. At 19°C and 22°C, hatch took 6.4 and 6.7 days respectively, and below 16°C, embryonic development of the *An. sinensis* cannot be completed. Hatching rates ranged from 63.68 to 90.88% (Wang et al., 1985; Li et al., 1994; Lai et al., 1995; Shen et al., 1995; Qu et al., 2000e) in different provinces in China. Hatching rates were lower under natural conditions compared to under experimental conditions, and there could be diapause in embryogenesis due to the low temperature (Zhao and Zhen, 1997). Larval breeding environments largely depended on the sites where females laid their eggs, and the oviposition locations were not stringent. The environment had no effect on the sex ratio (Zhou et al., 1988). The sex ratio was very close to 1:1 which meant that the natural quantity of males and females was equal.

The life cycle duration from the egg to the adult was a popular research topic. Eight studies between 1984 and 2000, indicated that the average duration of the life cycle ranged from 14 to 20 days (Wang et al., 1985; Zhu, 1989; Xu L. et al., 1991; Xu R. et al., 1991; Lai et al., 1995; Shen et al., 1995; Qu et al., 2000d,e). The development time also varied in different studies conducted in different regions of China. The longest time was 20.9 days in the Guizhou province (Wang et al., 1985), and the shortest time was 13.74 days occurred in the Henan province (Qu et al., 2000d). The time required for the development of *An. sinensis* was temperature dependent. Wang et al. (2010) documented a shortened duration as the temperature increased from 16 to 31°C. The life cycle duration were 30.7, 23.3, 15.5, 13.5, and 12.5 days at 19°C, 22°C, 25°C, 28°C, and 31°C respectively. *An. sinensis* can complete their development at temperatures as low as 16°C (Sun et al., 1994), but cannot develop successfully at temperatures > 31°C. In contrast, *An. gambiae s.s.* development was fastest between 28 and 32°C; adults did not emerged at <18°C or >34°C (Kirby and Lindsay, 2009). Development time from egg to adult was also largely temperature-dependent.

After completion of larval development, the pupation rate was generally high except in one study. Shen et al. (1995) documented a pupation rate of only 21.28–33.68%. Li et al. (1994) found that the larvae stage needed 8.16 days to develop and the mean duration for female larvae (8.54 ± 1.49) was longer than for males (7.78 ± 0.96). The larval stage fed on organic debris and microorganisms in the water including bacteria, protozoa, pollen grains, and fungal spores (Yuan et al., 1988). The distribution of young larvae in the water was similar in different rice field locations (Chen, 1990). The strong clustering habit of larvae weakened as they were growing, spreading from the center to around (Chen et al., 1990). Larval growth was influenced by many factors such as food types, food quantity, and larval density, etc. (Dong and Liu, 1990). Xu D. et al. (1988) indicated that development would be reduced if the larval density was too great.

Larvae of *An. sinensis* can successfully develop under a wide range of temperatures, whereas low winter temperature (usually after October in China) restricts the development of *An. sinensis* (Ding et al., 1991; Wu et al., 2005). In addition, Lai et al. (1995) noted that the time required for larvae development correlated with the time required for embryogenesis and hatching. If the time required for embryogenesis and hatching increased, the length of time required for larval development would decrease accordingly to keep the life cycle duration in balance of about 14 days, and vice versa (Lai et al., 1995).

The larval molted their exoskeleton four times before becoming pupae. *An. sinensis* remain in the pupal phase for about 2 days until the adults emerge and disperse to seek blood or nectar (Pan et al., 1984; Wang et al., 1985; Fengwen et al., 1988; Xu L. et al., 1988; Xu R. et al., 1991; Shen et al., 1995; Qu et al., 2000a). The eclosion rate from all the studies was higher than the hatching rate and the pupation rate (Shen et al., 1995; Zhong and Tan, 2000), indicating that death in premature stage mostly occurred during the larval stage. Compared to the high mortality during the larval stage, survival was higher in the pupal stage and during eclosion (Qu et al., 2000g,h). Like other mosquito species, the *An. sinensis* males often emerge first and form swarms, they cannot copulate until the genitalia rotate 180° which occurs in about 1 day. Females emerge subsequently, and then enter swarms to copulate in the air (Zhu, 1989).

*An. sinensis* is holometabolous insect and has four different developmental stages. The developmental parameters, such as number of eggs laid, hatching rate, pupation rate, and duration of the life cycle, can influence malaria transmission. A detailed understanding of *An. sinensis* biology pertaining to development could help in generating novel control strategies. Investigation of *An. sinensis* development may reveal mosquito-specific adaptations and could provide stage-specific targets for mosquito-borne infectious disease control.

### Longevity

The life expectancy of *An. sinensis* under natural condition was 5–7 days (Xu et al., 1987; Zhang et al., 1990; Qu et al., 2000e), which was shorter than the values measured under experimental conditions (Zhou et al., 1995) in which the constant nectar and blood were available. Female and male *An. sinensis* from different locations in China exhibited a significant difference in life expectancy. Qu et al. (2000a) observed average female and male *An. sinensis* life expectancies of 13.83 and 8.33 days, and maximum longevity was 32 and 14 days respectively (Qu et al., 2000f,i). Zhou et al. (1995) observed mean female and male life expectancies of 21.63 and 17.51 days, and maximum longevity was 51 and 46 days respectively. The results of the studies on *An. sinensis* life expectancy between female and male in different provinces indicated that the life expectancy of females exceeded that of males. In addition, temperature had a significant influence on the life expectancy of adult mosquitoes (Zhang et al., 1990; Xu L. et al., 1991). The average life expectancy in July was longer than that in August because the temperature in August was often higher than that in July (Qu et al., 2000d). In addition, Hu et al. (1988) showed that the average life expectancy was 48.6, 27.4, 23, 20.6, 14.8, and 11.1 days at 16°C, 19°C, 22°C, 25°C, 28°C, and 31°C. The longest life expectancy was at 16°C where individuals had very low metabolic rates (Pan et al., 1984; Hu et al., 1986).

### Gonotrophic Cycle

The mean gonotrophic cycle length for *An. sinensis* was 2.5 days (Liu, 1986; Xu et al., 1987, 1990; Wang et al., 1990; Li et al., 1994; Shi et al., 1996; Qu et al., 2000i). The shortest gonotrophic cycle for *An. sinensis* was 48 h (Wang et al., 1990) in Shanghai, while the longest was 2.65 days (Xu et al., 1987) in Fujian Province. The gonotrophic cycle for the different strains (Shanghai, Zhengzhou and Fujian strains) were similar (Qu et al., 2000b), but varied slightly related to the month (Zhang et al., 1990; Qu et al., 2000d), blood source (Su and Su, 1989), temperature (Xu and Zhang, 1988) and sunlight (Sun et al., 1987).

### Hibernation

Generally, *An. sinensis* begin to appear in late April or May and disappear in October. When the temperature related to the about 10°C, *An. sinensis* tended to seek sheltered places for hibernation. On the basis of studies on the hibernation of *An. sinensis* (Ma, 1954), together with studies conducted in Zhejiang, Henna, Hubei, and Jiangsu provinces etc. (Liu and Chen, 1959; You et al., 1964; Xue et al., 1990), *An. sinensis* hibernates in the adult stage. Both male and female adults were caught in cow sheds, mountain caves, and cellars. The mosquitoes caught in hibernation sites were found to be nulliparous and have sperm in the spermathecae. Much body fat accumulated during the winter months. In northern China, *An. sinensis* females hibernated in sheltered places from the end of October onward (Anonymous, 1973b; Chen et al., 1979). When the weather warmed the following spring, the hibernating mosquitos became active and fly off for the new life cycle (Anonymous, 1979).

### Flight Dispersal

*Anopheles sinensis* has limited dispersal with most adults staying close to their larval sites or habitats. Dispersal distances are generally less than 1 km, but longer passive dispersal by planes, ships, or other human devices could occur. There were relatively few studies on adult flight range or dispersal. Liu et al. (2011) studied the dispersal range of *An. sinensis* using a mark-release-recapture technique. They marked 3000 wild *An. sinensis* and recaptured mosquitos for ten successive days using light traps. The recapture rate was very low and most marked *An. sinensis* were recaptured within a 100 m radius of the release site. The maximum flight distance was 400 m.

## Bionomics

### Distribution

*Anopheles sinensis* has been recorded from Afghanistan, Myanmar, Cambodia, China, Indonesia, India, Japan, Korea, Malaysia, Singapore, Thailand, and Vietnam (Reid, 1953). It is distributed in 29 provinces within China and all regions except Xinjiang and Qinghai provinces (Lu et al., 1997). Xu and Feng (1975) published the first national distribution of the *Anopheles hyrcanus complex* in China including *An. sinensis*. The report primarily consisted of a brief survey of egg, larval, pupa and adult stages around the surveyed sites during 1962–1965. The results were limited to a short description of the main bionomic characteristics.

**FIGURE 3:**
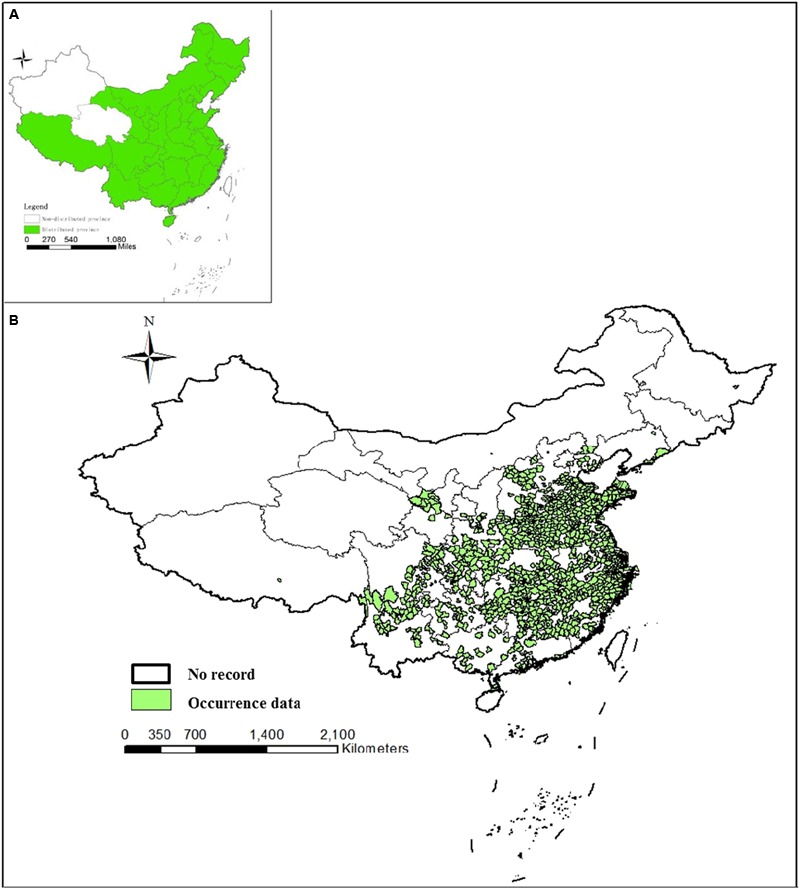
Distribution maps of *An. sinensis* in China. **(A)** Distribution of *An. sinensis* at province level. **(B)** Distribution of *An. sinensis* at county level based on occurrence data. Points have been georeferenced and digitized from publication maps using current departmental base map of China.

In the present review, an *An. sinensis* distribution map was produced by overlaying occurrence data from 332 source collected reports. *An. sinensis* was the mosquito taxon most commonly found and identified in China. However, this study did not document an extension to its previous distribution range. *An. sinensis* were found in 29 provinces and regions (**Figure 3A**) consistent with historical records. According to the analysis, *An. sinensis* has been recorded both on mainland (Chen, 1990; Pan et al., 2012) and on island areas (**Figure 3B**) (Zhang et al., 2001), and it was widespread across the plain areas and mountainous areas but more abundant in plain areas (Huang et al., 2015). *An. sinensis* was collected at 270 up to 2,300 m in elevation, but is generally restricted to elevations of 300–500 m (Liu et al., 1989). A more accurate, or a lower level distribution map is currently unavailable and the data currently consist of heterogeneous records that were not made on a national scale.

### Seasonal Population Fluctuation

In almost all areas in China, *An. sinensis* was found from July to December with a population peak in August. The population density of *An. sinensis* in cow sheds had a June peak in most places with a second small peak in late August or September. In Heilongjiang, Jilin, Liaoning and Xizang, *An. sinensis* was recorded only in August and September after which no specimens were found due to cold weather. In some warmer provinces like Yunnan, active *An. sinensis* were present in December but in relatively small numbers. In general, the collection data showed the seasonal abundance of *An. sinensis* fluctuated monthly (temperature-driven model) at various study sites during different study periods. *An. sinensis* emerged in April, developed into large populations from July to December with a peak in August.

### Habitats

#### Larval Habitats

*Anopheles sinensis* larvae were found in diverse habitats. Examples of major habitats include rice-fields and ponds (Liu et al., 1962). The larvae were also collected in irrigation channels, abandoned wells, ground pools, and pools beside rivers, marshes, stream margins, ditches, seepages, shallow ponds, sumps, hoof footprints, and wheel tracks. The larvae have also been found in polluted pools and cesspits (Cai, 1959). Environmental factors associated with larval habitats have been studied. The available data (Anonymous, 1973b; You and Xu, 1979; Chen, 1990) observed that the breeding sites of *An. sinensis* were located within 50–200 m from irrigation wells, human or livestock settings, and the larvae were most often found in water bodies with abundant aquatic vegetation. Physicochemical properties tests of habitats showed that *An. sinensis* larvae had high adaptability to variable water quality (Wang, 1979).

#### Indoor and Outdoor Habitats

The preferred indoor habitats (resting habits) for *An. sinensis* were mainly cow sheds, pigpens, sheepfolds and human dwellings (Cai, 1959; Ouyang et al., 1992; Yi et al., 1998), especially when they are seeking hosts for blood meals. About 61% of resting *An. sinensis* was captured from cow sheds, 37.7% from human dwellings, and 0.74% from spare houses (Cai, 1959). A report from Jaingsu province found that the average number of *An. sinensis* in livestock sheds was 49 times greater than that in human dwellings (Gao et al., 2004). Both studies found a higher tendency for resting mainly in cow sheds, although other resting places can be selected.

Outdoor habitats for *An. sinensis* were mainly grass or leaves growing near or along rice fields and streams when they are not actively seeking hosts or oviposition habitats. Typical plants were rice near permanent water or subject to regular irrigation, or sweet potatoes, vegetables, shrubs and weeds around human residences and livestock sheds. Other potential outdoor resting sites were soil cave and ravines with damp, dark and humid environments (He et al., 1962). Most *An. sinensis* rested at heights of 5–15 cm above the ground on the trunk of a plant or under leaves.

The habitats for *An. sinensis* varied among the different regions, and were under the influence of the local biotope. There was evidence for various habitats with diverse geography. For instance, *An. sinensis* on Hainan Island tended to rest on the grass or other nearby vegetation after a blood meal and disperse before dawn, perhaps seeking more secluded places to rest. *An. sinensis* in Guangdong, Guangxi, and Shanghai were more likely to stay in structure for a period after feeding, usually a human house or cow shed (Liu, 1962). This difference may be the result of different natural environments in these regions. For example, Hainan Island has a tropical climate and dense vegetation. In contrast, there is relatively less vegetation in other regions but they have more buildings with solid walls. The perennial impact of the external environment may have altered feeding habits.

The factors affecting *An. sinensis* habitat were more complicated. Despite clear evidence that the longitude and latitude have an influence on *An. sinensis* habitats, other factors such as temperature, rainfall, human activities are also involved. Human and animal activity seemed to play a critical role in habitat selection. Local residents sleeping outdoors, degree of vegetation cover, and grazing habits of livestock were all important in habitat preferences of *An. sinensis*.

### Biting Habits and Feeding Preferences

Female *An. sinensis* fed throughout the night but were most active from sunset to midnight. Peaking feeding activity apparently occurred at different times depending on locality and habitats. Cai (1959) showed that *An. sinensis* in human dwellings fed from dusk to dawn, with two peaks one between 8:00 pm and 9:00 pm and another at 1:00 am. In cow sheds, only one peak occurred at 8:00 pm. Chen et al. (1979) observed peak activity in Henan province occurring between 8:00 pm to 9:00 pm. Peak activity in Zhejiang province was from 9:00 pm to 10:00 pm (Zhang et al., 1990). Similar peak activity has been recorded in several other areas of China and during different time periods. Considering that these data were somewhat dated, we examined the most recent studies conducted between 2013 and 2014 in Hunan Province (Zhu et al., 2015) and in 2012 in four counties in Yunnan Province (Zhang et al., 2015). By contrast, although the results differed in the bait method, selected localities, weather conditions and other factors, the similar peak activity, either one peak after the sunset (between 8:00 pm and 10:00 pm) or with a second peak before dawn (between 1:00 am and 2:00 am) could be observed in different localities.

Under normal circumstances, *An. sinensis* females are facultative feeders but relatively more zoophilic. *An. sinensis* preferred to feed on livestock in the presence of both humans and their preferred animal hosts (buffalo and cattle), and they were prone to be found inside livestock corrals. Proportion tests on blood meals to detect feeding preferences revealed that most *An. sinensis* were zoophilic. However, the females also fed on the blood of whatever vertebrates are available in the vicinity, and sometimes they readily fed on humans. *An. sinensis* mostly feed on large animals such as cows, buffalos, pigs, horses, donkeys, mules, and goats. Among them, cows were the most attractive animal. Two studies (Malaria Group, 1984; Qian et al., 1984) between 1979 and 1981 showed that the human blood proportion was 3.52 and 2.60% respectively in captured *An. sinensis*. Hu et al. (1988) also found a low percentage of human blood (2.90%) in *An. sinensis* compared with cow blood. So *An. sinensis*is is facultatively anthropophilic but prefers to be zoophilic.

### Vector Competence

Malaria parasites must undergo development within the mosquito before they are infectious to humans. *An. sinensis* is a *P. vivax* malaria vector in China, Indonesia, Japan, and South Korea (Rueda et al., 2010). In China, Wang et al. (1982) and Qu et al. (2000c) studied *P. vivax* development in *An. sinensis*. Within 15–20 min after mosquito ingestion, the male (microgametocytes) and female (macrogametocytes) gametocytes could be detected in the mosquito’s midgut. The fertilization event produced a zygote, then developed into an ookinete in as short as 50 min and fully developed within 26 h. The ookinete traversed the peritrophic membrane of the midgut, crossing the midgut epithelium, entered the basil lamina, and formed an oocyst in 48 h. Over a period of 3–7 days, after oocyst maturation was completed, the oocyst ruptured to release multiple immature sporozoites into the haemolymph. The sporozoites then migrated to the salivary glands in 7–8 days. The *Plasmodium vivax* cycle begins again when the female mosquito takes a blood meal, injecting the sporozoites from its salivary glands into the human bloodstream.

Vector competence is an indication of mosquitoes ability to obtain a disease agent (microorganism, such as parasite, arbovirus etc.) from a reservoir host and then transmit the infectious agent to another susceptible host (Min et al., 2002). Vector competence for malaria is evaluated by the susceptibility of Anopheline species to malaria parasites and the ability to transmit a susceptible host (Beier, 1998). Generally, malaria vector competence is determined through either observation of sporozoites in the salivary glands of field-caught mosquitoes or infection experiments using laboratory reared mosquitoes. *An. sinensis* natural infection has been extensively studied (Anonymous, 1973a, 1975; Gou et al., 1998; Zhou et al., 2005) and had sporozoite rates ranging from 0.00 to 0.33% in the salivary glands under microscope. A null sporozoite rate was detected in Hunan, Guangxi, and Shanghai (Malaria Group, 1984; Wang et al., 1987, 1990), a 0.16% rate was found in Pucheng county, Fujian province (Xu et al., 1990), and 0.33% in Xuzhou, Jiangsu province (Qiu and Zhao, 1978). *An. sinensis* is a confirmed malaria vector in China and has been reported naturally infected with malaria parasites in many provinces. However, the results of published studies by dissecting the salivary gland of field *An. sinensis* only reported the positive sporozoite rates, but failed to identify the Plasmodium species until the sporozoite ELISA kit was applied.

Experimental infection of *An. sinensis* against *P. vivax* has been done in different provinces at different times. Hu et al. (1981) found that *An. sinensis* oocyst rates (23.66%) were significantly higher than sporozoite rates (6.21%) in Shandong province and determined that 25–28°C was the best temperature range for *P. vivax* sporozoite multiplication within *An. sinensis*. Wang et al. (1982) found much higher *P. vivax* oocyst rates (38.10–95.90%) and sporozoite rates (21.90–50.80%) under experimental condition both in two *An. sinensis* strains in Henan province. Xiangkun et al. (1991) observed the highest *P. vivax* oocyst rates and sporozoite rates in the *An. sinensis* Guizhou strain (81.22–100.00%; 86.36–100.00%) and the Shanghai strain (85.71–100.00%; 91.67–100.00%) under experimental infection respectively. These findings suggest that there can be a large difference in the *An. sinensis* experimental infection rate depending on both the strain of mosquito and the parasite.

The susceptibility of different *An. sinensis* strains to a single *P. vivax* strain, together with the susceptibility of a single *An. sinensis* strain to different *P. vivax* strains was studied by Zheng et al. (1986), Xu and Ye (1987), and Zhu (1989). Xu et al. (1987) reported that the susceptibilities of Shanghai and Guangxi *An. sinensis* strains against the Guangxi *P. vivax* strain were low (0.00% and 12.3%), but significantly higher against Hainan *P. vivax* strain (36.40% and 43.60%). Zhu (1989) observed that the susceptibility of the Simao *An. sinensis* strain against *P. vivax* was significantly lower compared to the Shanghai *An. sinensis* strain. In contrast, Zheng et al. (1986) reported similar sporozoite rates in four different *An. sinensis* strains (Changsha, Shanghai, Zhengzhou, and Wuhan strain) against two *P. vivax* strains. Vector competence may vary among different *An. sinensis* strains for different *P. vivax* strains but the reasons for this are not clear.

In addition to the mosquito infection experiments, the researchers also investigated the correlation of infection rate with the patient status (Shi et al., 1983b; Qu et al., 2000c), parasite density in donor blood (Zheng et al., 1986), long incubation period strains (Xu and Ye, 1987), course of the disease (Zheng et al., 1986; Xu and Ye, 1987), and relapse or recrudesce (Zhu, 1993; Zhu et al., 2007, 2013), etc. Shi et al. (1983b) showed that *An. sinensis* sporozoite rate of the long incubation period *P. vivax* strain was higher (61.0%) than that in short incubation period *P. vivax* strain (33.3%), and infection rate of *An. sinensis* was correlated with the density and sex ratio of gametocytes. Li et al. (1998) reported that infection rate of *An. sinensis* for *P. vivax* under experimental conditions increased with parasite density and stage of the disease. *An. sinensis* showed a high infection rate in relapse or recrudesce malaria cases. Li et al. (1998) and Zhu et al. (2007) both reported infection rate and oocyst rates of *An. sinensis* to *P. vivax*. The blood resources from patients in the fever stage were lower than those in non-fever stage patients. Interestingly, Zhu et al. (2007) found that sporozoite rates were higher in the *An. sinensis* group fed on fever stage patients in the same study. However, in another study, Zhu et al. (2013) reported that the gametocytemia, asexual parasitemia, and ratio of macrogametocytes to microgametocytes, did not correlate with either oocyst or sporozoite infection, while in the oocyst-positive mosquitoes, there was a correlation between gametocytemia and the average oocyst number.

*Anopheles sinensis* was refractory to *P. falciparum* in Thailand (Rongsriyam et al., 1998). How about this situation in China? *An. sinensis* susceptibility to *P. vivax* versus *P. falciparum* in Guangxi province showed average infection rates of 59.80% (oocyst rate) and 12.80% (sporozoite rate) compared to no infection with *P. falciparum* (Xu et al., 1983). However, studies conducted by Shi et al. (1983a) and Liu et al. (1984) indicated that *An. sinensis* could be infected with *P. falciparum* but at a relatively low rate. Shi et al. (1983a) found that *P. falciparum* oocyst rates ranged from 2.14 to 8.6%, and sporozoite rates ranged from 0.00 to 3.54%. Liu et al. (1984) observed an average *P. falciparum* oocyst rate of 11.30% and average sporozoite rate of 3.00%. These provide evidence that *An. sinensis* in China is not totally refractory to *P. falciparum*. Vector competence of *An. sinensis* is thought to be significantly higher for *P. vivax* compared to *P. falciparum.*

In summary, *An. sinensis* infection experiments showed variable sporozoite infection rates with *P. vivax* (6.21–100.00%) and *P. falciparum* (0.00–3.54%). The presence of a natural infection sporozoite rate together with the relatively high value of both oocyst and sporozoite rates under laboratory infection experiments supports the potentially high vector competence for *P. vivax*. Although *An. sinensis* could be infected by *P. falciparum* in some studies, dissection of salivary glands in field *An. sinensis* failed to reveal the presence of *P. falciparum* sporozoites. These data support the conclusion that *An. sinensis* is not a vector for *P. falciparum* malaria in China.

### Vectorial Capacity

Vectorial capacity is a measurement of the efficiency of vector-borne disease transmission. In addition to competence, vector capacity includes a variety of other essential traits, i.e., host preferences, biting rate, mosquito population density, longevity, and etc. The malaria transmission intensity can be characterized as the vectorial capacity (C) formulated by Macdonald (1957) and Garrett-Jones (1964), which is defined as:

$$C=\frac{\text{m}\alpha^{\text{2}}p^{n}}{-\ln(p)}$$

where *ma* is the human biting rate (the number of female mosquitoes per person per night); *a* is the human-biting frequency per day (daily probability of a human host being bit by a vector, which is indirectly estimated as the human blood index divided by duration of gonotrophic cycle; *n* is the length of the sporogonic cycle; *p* is the daily survival rate of the vector.

In China, the *An. sinensis* vector capacity was measured and compared by investigating of *An. sinensis* at different times and in different provinces (**Table 1**). The first and most recent reports of *An. sinensis* capacity were published in 1983 and 2015, the results showed that VC was 0.24 and 0.15 respectively (Song, 2015). The vector capacities of *An. sinensis* varied from north to south in China, and were correlated with the season (Yao et al., 2002). Pan et al. (2008) founded that the vector capacity of *An. sinensis* in Huizhou and Zhuhai, Guangdong province was 0.25 and 0.66, respectively. Zhao et al. (1996) and Yu et al. (2014) reported that the vector capacities of *An. sinensis* were 0.01 and 0.15 in Sichuan and Shandong province (north), respectively. Differences observed in the vectorial capacity of *An. sinensis* may be due to variation in ecological habits or genetic background in different localities. The overall low vectorial capacity of *An. sinensis* may be diminished by tendencies for zoophilic feeding behavior in some study sites. In addition, vectorial capacity of *An. sinensis* could also serve as an indicator for annual parasite index (API) or malaria prevalence (Yang and Tian, 1994; Liu et al., 1995; Qian et al., 1996).

**Table 1:** Vectorial capacity (VC) and entomological inoculation rate (EIR) of *An. sinensis* in China.

Reference	Location	*ma*	HBI	GC	*a* (HBI/GC)	*n*	*p*	*C*	SR (%)	EIR (*ma^∗^* SR^∗^coefficient)
Anonymous, 1975	Shangqiu, Henan	13.80	NA	3.00	NA	10.50	0.85	NA	0.09	0.24
Liu et al., 1983	Zhejiang	6.90	NA	NA	NA	NA	0.85	0.24	NA	NA
Liu et al., 1984	Shucheng, Anhui	NA	NA	NA	NA	NA	NA	NA	0.19	0.00
Qian et al., 1984	Pixian, Jiangsu	NA	0.10	2.50	0.04	NA	0.86–0.88	NA	NA	NA
Station, 1985	Guidong, Hunan	0–77.25	NA	NA	NA	NA	NA	0–2.03	NA	NA
Hu, 1986	Yunnan	NA	NA	NA	NA	NA	NA	0.01	NA	NA
Liu et al., 1986	Junlian, Sichuan	0.41	0.05	2.50	0.02	10.00	0.88	0.02	0.00	0.00
Wang et al., 1987	Gunagxi	0.03–2.02	NA	NA	0.02–0.05	NA	NA	0.00–0.26	NA	NA
Xu et al., 1987	Fujian	8.10	0.08	NA	0.03	9.80	NA	0.19	NA	NA
Mo et al., 1988	Gunagxi	13.60	0.30	NA	NA	NA	NA	0.08	0.19	NA
		0.80	0.02					0.02	0.00	
Xu L. et al., 1988	Fujian	8.10	NA	NA	0.03	9.80	NA	0.28	NA	NA
Zhang et al., 1989	Yunnan	1.40	0.05	2.50	0.02	NA	0.87	0.03	0.00	0.00
Wang et al., 1990	Shanghai	9.14	0.06	2.00	0.03	NA	0.86	0.35	0.00	NA
Xu et al., 1990	Fujian	8.10	NA	2.50	0.03	9.80	0.84	0.26	0.16	NA
Ouyang et al., 1992	Hunan	NA	NA	NA	NA	NA	NA	0–2.7	NA	NA
Yang et al., 1994	Yanhe, Guizhou	32.90–76.50	NA	3.00	0.04	NA	NA	0.42–14.94	NA	NA
Liu et al., 1995	Ganyu, Jiangsu	0.25–2.38	NA	NA	0.00–0.03	NA	0.85–0.90	0.00–0.12	NA	NA
Nie et al., 1996	Longli, Guizhou	2.50	0.02	NA	0.01	14.51	0.82	0.01	NA	NA
		1.31	0.02		0.01	14.82	0.85	0.01		
Shi et al., 1996	Deqing, Zhejiang	NA	0.04	2.50	0.02	8.50	0.87	0.04	NA	NA
Zhao et al., 1996	Jining, Shandong	4.07	0.13	2.50	0.05	10.00	0.82	0.15	NA	NA
Zhang and Zhang, 1997	Tengchong, Yunan	1.33	0.03	2.50	0.01	NA	0.78	0.00	0.00	NA
Lang et al., 2000	Taizhou, Zhejiang	3.08	NA	3.67	0.01	11.39	0.79	0.01	NA	NA
Feng et al., 2002	Qionglai, Sichuan	0.12	NA	NA	0.02	NA	NA	0.01	NA	NA
Yao et al., 2002	Haining, Zhejiang	9.85	NA	3.70	0.01	9.50	0.81	0.08	NA	NA
Gao et al., 2004	Changzhou, Jiangsu	NA	0.00	2.00	NA	NA	NA	0.00	NA	NA
Luo et al., 2006	Yiwu, Zhejiang	15.36	NA	3.67	0.02	8.54	0.87	0.73	NA	NA
Zhang X. et al., 2006	Wenzhou, Zhejiang	7.24	0.06	NA	0.02	NA	NA	0.22	NA	NA
Wang et al., 2007	Mengcheng, Anhui	11.19	0.18	2.50	0.07	9.79	0.83	0.15	NA	NA
Li et al., 2008	Fenghua, Zhengjiang	2.87	0.04	2.50	0.02	10.84	0.91	0.18	NA	NA
Pan et al., 2008	Huizhou, Guangdong	5.01	0.48	NA	0.13	NA	NA	0.25	NA	NA
	Zhuhai, Guangdong	4.31	0.44		0.12			0.66		
Pan et al., 2012	Huaiyuan, Anhui	6.10	0.67	2.50	0.27	10.00	NA	0.77	NA	NA
	Yongcheng, Henan	5.90	0.64	2.50	0.26	10.00		0.55		
Yang et al., 2013	Shuangliu, Sichuan	1.93	0.05	3.50	NA	NA	NA	0.02	NA	NA
Yu et al., 2014	Chengdu, Sichuan	1.20	0.05	3.50	NA	NA	0.85	0.01	NA	NA
Song, 2015	Xiangshan, Zhejiang	2.64	NA	3.67	0.03	10.41	0.88	0.12	NA	NA

*ma, Human biting rate; HBI, Human blood index; GC, Gonotrophic cycle; SR, Sporozoite rate; EIR, entomological inoculation rate; NA, not available.*

Vectorial capacity includes a number of factors. Human blood index (HBI) and human biting rate (*ma*) are important indicators for vector capacity, and would determine the capacity to transmit malaria protozoa. Two studies reported that the vector capacity of *An. sinensis* was highest in July or August corresponding to the highest human biting rate (Station, 1985; Yao et al., 2002). Another study conducted by Qian et al. (1996) compared the vector capacity of *An. sinensis* between the 1970s and the 1990s, and found a significant decrease both in human biting rate and vector capacity. In other studies conducted in different provinces a positive correlation between the HBI, human biting rate and vector capacity (Liu et al., 1995; Zhang X. et al., 2006).

Other indicators such as duration of the gonotrophic cycle (GC), length of the sporogonic cycle (n), and the daily survival rate of the vector (*p*) generally have stable values corresponding to the mosquito species. In different studies, the gonotrophic cycle of *An. sinensis* in China was 2.5–3.7 days, the length of the sporogonic cycle was 8.5–14.8 days, and the daily survival rate of the vector was 0.77–0.91. Given that the *P. vivax* incubation time in *An. sinensis* was of 9–14 days, *An. sinensis* would require two to three blood meals before becoming infectious agents. In addition, calculation of daily survival rate requires introduction of another parameter–the parity rate. Studies by Gao et al. (2004) found the mean parity rates of *An. sinensis* samples collected over years from Hunan province to be 42.86%. The parity rates varied in different habitats, months, indoor and outdoor environments, daytime and night, as well as before midnight and after midnight (Wang, 2013). This variation may be attributed to numbers of emerging mosquito, the mortality rate of larvae and sampling methods (Qian et al., 1984).

The vectorial capacity measures efficiency of pathogen transmission, but EIR is a measure of malaria transmission intensity. It is usually interpreted as the number of infectious bites per human during a season or annually (usually annually) and is referred as a more direct way to measure transmission risk. However, although studies (Anonymous, 1975; Liu et al., 1984; Liu et al., 1986; Zhang et al., 1989) have provided estimates of EIR values ranging from 0.000031 to 0.24 (**Table 1**), which is conventionally computed by taking the product of biting rate multiplied by the sporozoite rate. The data still made it difficult to investigate the accuracy and precision of EIR because sporozoite rates were exceedingly low (<0.19%), while appropriate values ranging from 1 to 20% are generally suggested for EIR determination in Africa.

## Molecular Biology

### Molecules Revealed by Genome and Transcriptome

Scientists have unveiled the genomes of two *An. sinensis* strains from Korea^1^ and China (Zhou et al., 2014). The genome of *An. sinensis* (China strain) was fully sequenced in 2014. *An. sinensis* has six chromosomes representing two three-chromosome sets. The genome size was estimated 220.78 Mb, coding for 16, 766 genes. Approximately 14% of the putative genes were orthologous with genes in 235 known biological pathways. The genome had 3, 972 gene clusters containing 11,300 genes that were common to the genomes of three previously sequenced mosquito species, *An. gambiae, An. aegypti*, and *Cu. quinquefasciatus*. Gene orthology prediction revealed a total of 4,727 orthologous genes were shared among the mosquitoes (Zhou et al., 2014). Analysis of these orthologous genes revealed the most gene-enriched domain and family were peptidase, while KEGG pathway revealed that genes were most enriched in metabolic pathways. The results indicate that functions such as feeding behavior are central to mosquito biology. In addition to protein-coding genes, 41 microRNA, 348 tRNA and 2017 rRNA genes have also been identified.

A comprehensive reference transcriptome of *An. sinensis* sampled from different developmental stages of egg, larva, pupa, and adult was sequenced by Chen et al. (2014). Approximately 51.6 million clean reads were obtained, and these were assembled into 38,504 unigenes. Among them, 98.4% (37,884/38,504) of unigenes could be mapped onto the *An. sinensis* reference genome, and 69% (26,650) of the unigenes could be annotated with known biological functions. In addition, a total of 8,057 expressed sequence tags (ESTs) were assigned to GO and KEGG annotation. A large number of ESTs were restricted to metabolic pathways, biosynthesis of secondary metabolites, and microbial metabolism. The study also found that 2,131 ESTs were differentially expressed between deltamethrin resistant and deltamethrin susceptible mosquitoes collected from the same field site. Further, more than 2,400 microsatellite markers have also been identified (Zhu et al., 2014). These studies would definitely enhance knowledge on the *An. sinensis* and lay an important foundation for further functional analysis so as to provide new tools for future malaria vectors control.

### Molecules Identified by Experiments

A compendium of molecules are involved the interaction of the Plasmodium parasites within their host vectors (Sreenivasamurthy et al., 2013), however, little is known about the analogous process in *An. sinensis*. The first gene of *An. sinensis* was studied by Zhi et al. (2002) who attempted to clone the defensin gene (immune gene) from the main mosquito vectors of China. Sequence analysis showed that the amplified fragments from *An. sinensis* were homologous to the reported defensin sequence of *An. gambiae*. Since then, newer research has focused on cloning the defensin gene, prokaryotic expression and the product activity (Yan et al., 2005; Zheng et al., 2005). The complete defensin gene was cloned by Zhang Y.J. et al. (2006). It has a total length of 2,256 bp, including the 5′ and 3′ UTR fractions and two exons separated by an 85 bp intron. The entire cDNA sequence of *An. sinensis* defensin gene was 324 bp; its ORF encoded 107 amino acids, and mature peptide had 40 amino acids residues. Then, another immune gene, partial SRPN14 gene of *An. sinensis*, was identified and characterized (Feng et al., 2015). The SRPN14 gene was found to be located on 2L: 23C of salivary gland chromosomes of *An. sinensis* by *in situ* hybridization, which had 77% (nt) and 88% (aa) similarities with *An. gambiae*. Several mosquito proteins have been involved in regulating the maturation of malaria parasites. For example, HSP40 was isolated from *P. yoelii* infected *An. sinensis* and its full-length cDNA of 1,159 bp with an ORF of 1,014 bp, encoding 337 amino acids was amplified (Li Y. et al., 2010). An analysis of gene expression throughout development has been conducted for *An. gambiae* (Strode et al., 2006). In *An. sinensis*, several important homologous genes have been identified (**Table 2**). Liu B.Q. et al. (2016) found that there were four CPF (cuticular protein family) gene families in *An. sinensis* genome. Analysis of these CPFs in *An. sinensis* revealed that the full-length cDNA sequences ranged from 531 to 1,001 bp and coded for 148 to 345 amino acids. These AsCPFs contained a 44-amino-acid conserved region and a C-terminal region, which were secretory proteins with signal peptide sequences except for AsCPF2 that was a non-secretory protein and lacked a signal peptide sequence but contained a transmembrane region. Qiao et al. (2016) studied on the role of expression and regulation of TH (the initial enzyme in the melanin pathway) on specific physiological processes during mosquito development by silencing of AsTH. Significant disruption of cuticle in experiments strongly suggested that TH was essential for pupae tanning and immunity in *Anopheles*.

**Table 2:** dentified published molecules of *An. sinensis* in China.

Molecules	Accession number	Functions involved	Reference
Defensin	DQ002892	Immunity	Zhi et al., 2002; Yan et al., 2005; Zheng et al., 2005; Zhang Y.J. et al., 2006
Aquaporin (AQP)	NA	Biological process	Tang et al., 2012
CYP6P5	KF358704	NA	Che et al., 2014
CYP4G17	NA	NA	Yan et al., 2015
SRPN14	NA	Immunity	Feng et al., 2015
Detoxification supergene families	NA	Insecticide resistance	Zhou et al., 2015
Haem oxygenase (HO-1)	NA	NA	Zhi et al., 2015
Tyrosine hydroxylase (TH)	NA	Immunity/Biological process	Qiao et al., 2016
Odorant-binding protein (OBP)	NA	Biological process	Qin et al., 2014; He et al., 2016
Iron responsive element binding protein 1 (IRE_BP1)	NA	Insecticide resistance	Wang et al., 2015
Cuticular protein family (CPF)	NA	Biological process	Liu B.Q. et al., 2016
Heat shock protein 40 (HSP40)	HM013840	Immunity/molecular chaperones	Li Y. et al., 2010

*NA, not available.*

In addition, AQPs (water channels) are integral membrane proteins in biological cells. Some AQPs were abundantly expressed in Malpighian tubules of *An. gambiae*, and reduced expression could increase mosquito survival in dry environments (Liu K. et al., 2016). Tang et al. (2012) identified a full-length cDNA of AQP from *An. sinensis*, which consisted of 762 bp coding for 253 amino acids, with a predicted molecular mass of 63.2 kD. The AsAQP shared 76 and 78% identities with AQPs of *Cu. quinquefasciatus* and *Ae. aegypti* AQPs, respectively. To analyze the differential expression of iron responsive element binding protein genes in different strains of *An. sinensis*, Wang et al. (2015) identified partial sequences of IRE_BP1 by two-dimensional electrophoresis followed by corresponding PCR applications. They found that the IRE_BP1 gene expression in resistant *An. sinensis* strains was 9.42 times than that of susceptible strains by fluorescence quantitative PCR. The IRE_BP1 may be useful as a resistance control target and gene detection indicator.

The recently published *An. sinensis* genome and transcriptome provide an opportunity for advanced study of gene products. Based on the comparison between OBP conserved motifs with *An. gambiae*, He et al. (2016) identified 64 putative odorant-binding protein genes (OBP) in the genome of *An. sinensis*. The authors also investigated motif conservation, gene structure, genomic organization and classification. *An. sinensis* OBP genes were classified into three subfamilies, some genes might have originated from a single gene through a series of historic duplication events. Zhou et al. (2015) found 174 detoxification genes by analyzing *An. sinensis* genome, including 93 cytochrome P450s (P450s), 31 GSTs, and 50 choline/carboxylesterases (CCEs). Based on a combination analysis of available *An. sinensis* transcriptome, several candidate genes overexpressed in a deltamethrin-resistant strain were identified as belonging to the CYP4 or CYP6 family of P450s and the Delta GST class. Che et al. (2014) identified CYP6P5 gene in *An. sinensis* and analyzed its bioinformatics characteristics. The entire sequence was 1, 583 bp long with an ORF of 1, 527 bp, encoding 508 amino acids. Phylogenetic and similarity analyses of amino acid sequences showed that *An. sinensis* CYP6P5 had the closest phylogenetic relationship to the CYP6P5s of *An. funestus* and *An. gambiae*, with similarity values of 89.4 and 89.0%, respectively.

Although, the current literature on molecular biology was systematically reviewed, significant gaps in knowledge of *An. sinensis* basic biology remain. For instance, recent advances in molecular biology have resulted in development of a genetically modified mosquito species for the purpose of population control (Lounibos, 2004; Christophides, 2005). More inspiringly, mosquito gut microbiota have emerged as a novel target to modulate homeostasis in greater depth and to develop new paradigms for disease transmission control (Rodgers et al., 2017). Therefore, studies on vectors and their molecular biology remain important to develop possible applications for more effective vector control.

## Discussion

This report updates available knowledge on *An. sinensis* and characterizes biology, bionomics and molecules research in China through an extensive review gathered in the country from 1954 to 2016. Most of the literatures on biology and bionomics were during the early time of high incidence periods in China. In contrast, there was little recent entomology research focused on the ecology and life history study in recent years. Research conducted before 1970 was generally limited and sporadic. Entomological research increased following two events. One event occurred in 1985 and it was the start of a nationwide malaria control program activated by two decades of outbreak and pandemic transmission between 1960 and 1979 (Yin et al., 2014). Another event started in 2003 after GFATM began to support malaria control campaign in the People’s Republic of China (Wang et al., 2014). The first molecular study on *An. sinensis* was published in 2002, but there are still few publications on gene functions and molecular mechanisms, despite a large numbers of molecules being found using omics tools.

A detailed summary of biology information on *An. sinensis* was presented and represents the accumulation of many years’ studies in China. Before molecular tools were developed, the identification of *An. sinensis* and its sibling species depended on morphological characteristics (Reidenbach et al., 2009), which were sometimes undependable and likely to misidentify sibling species. For instance, a narrow-ovate form of *An. sinensis* was first described as a new species but later identified as *Anopheles lesteri* (Makhawi et al., 2013). PCR assays based on molecular markers have been developed to accurately distinguish in this species complex (Fanello et al., 2002) in 1990s. *An. sinensis* has been successfully identified from *Anopheles hyrcanus* complex based on specific DNA nucleotide differences in the sequences of the second internal transcribed spacer (ITS2) of the ribosomal DNA (rDNA) (Ma and Xu, 2005). Morphological characteristics together with nucleotide differences have enabled accurate identification of *An. sinensis* from *Anopheles hyrcanus* complex, which is essential for more precise knowledge of this species.

*Anopheles sinensis* has a complex life cycle. Current knowledge of the *An. sinensis* life cycle is basically complete and sustained by extensive field investigations and laboratory rearing experiments. Most egg laying of *An. sinensis* occurs at the night following digestion of a blood meal, however, exceptions to this behavior have also been noted. For example, egg laying is sometimes observed in the daytime (Yusheng and Xingbang, 1958). The timing and amounts of oviposition depend on local conditions, blood-feeding, and the season. Various experimental factors, such as photoperiod, substrate and bacteria, were also involved (Zhao et al., 1991; Pan et al., 1993; Li and Tang, 2010). Oviposition time and location may have important consequences for vector population dynamics and epidemiology.

Knowledge of the different stages of *An. sinensis* should enhance the efficacy of control strategies. *An. sinensis* larvae lie horizontally at the water surface and feed on microorganisms, algae, protozoa, and organic detritus. The rate of larval development is temperature dependent and correlated to the time required for embryogenesis and hatch. The pupa stage usually lasted about 2 days, and this may be extended or shortened related to temperature. The survival rate of *An. sinensis* pupae was highest compared to the other development stages. There was a difference between male and female sexual maturity at the time of emergence. Males usually emerge 1∼2 days before the females. *An. sinensis* has a limited flight range of 1 km; the species tend to stay near their breeding sites. Marked-release-recapture experiments indicate that they normally do not migrate long distances.

Studies on male and female *An. sinensis* life expectancy among different geographical strains indicated that male life expectancy was shorter than female. These results were consistent with the results of Suleman’s and Suman’s on *Cu. quinquefasciatus*. Shorter male life span may be a kind of biological rule for male and female mosquitoes. High temperatures and low humidity can also reduce *An. sinensis* longevity while sufficient food supply can extend the longevity, a result that is especially obvious under experimental conditions. *An. sinensis* overwinter as adult females. Usually, the females use the lipid reserves for overwintering survival in protected shelters when the temperature is less than 10°C.

Several studies described the complexity of *P. vivax* development in *An. sinensis.* The *P. vivax* gametocytes completed fertilization soon after mosquito blood-feeding on an infected host. They transformed to ookinetes and oocysts, and began to invade the salivary glands at 7∼8 days. Under experimental conditions, the sporozoite rates were lower than the oocyst rates (Wang et al., 1982; Qu et al., 2000c). Sporozoite rates were positively correlated with oocyst rates, and different *An. sinensis* strains showed different sporozoite rates and oocyst rates (Wang et al., 1982). The mean duration of the gonotrophic cycle for *An. sinensis* was 2.6 days. Assuming a *P. vivax* incubation time in mosquitoes of 7–8 days, *An. sinensis* would require three to four blood meals before being capable of transmitting sporozoites.

In the context of malaria elimination, the value of understanding the bionomics of *An. sinensis* and its role on malaria transmission is critical for malaria reduction. Distribution maps of *An. sinensis* in China were produced by combining current knowledge of *An. sinensis* distribution, and occurrence data from open access, and published papers. The occurrence data resulted in the collation of *An. sinensis* occurrence from 343 records across 29 provinces. The map was limited by the available data and bias sampling on original data acquisition. The maps can still serve as an accurate representation of the ranges of *An. sinensis* in China. However, to increase knowledge of the biology and bionomics of the mosquitoe in China, it is important to extend entomological surveys to unexplored northern regions which may represent its northern distribution boundary. A better understanding of the full distribution range of *An. sinensis* and maps which could predict the distribution trend by combining climate change models is highly desirable.

Field studies on larval presence of in water bodies indicate that *An. sinensis* can select a wide range of water bodies in which to lay their eggs. These studies implied that larvae habitats were heterogeneous in form, space, and physical features. Indoor and outdoor habitats for adult *An. sinensis* were also very heterogeneous. This could be partially explained by the differences in the investigation periods and the methods used in *An. sinensis* collection, such as larval collection tools, light traps, and human baited trap.

Adult *An. sinensis* have been incriminated as a natural and experimental malaria (*P. vivax*) vector in China, Indonesia, Japan, and South Korea (Ree, 2005). The known published data on *An. sinensis* host selection in China indicated that the degree of anthropophily of *An. sinensis* was low, and it was generally concluded that *An. sinensis* is not an effective malaria vector, especially in northern China. However, since 1960, uninterrupted malaria cases have occurred in central China, where *An. sinensis* is the only vector in most areas, thus it is difficult to explain why *An. sinensis*is appears to be an effective malaria vector in these areas. According to a study in Henan province, *An. sinensis* not only fed on human blood, but the biting frequency was higher and natural infection of *P. vavix* was also present. The evidence indicated that *An. sinensis* served as the major vector for endemic malaria in these regions. This suggests that malaria transmission competence differs from south to north, and this is also supported by variable vector capacity. It was concluded that the vivax malaria transmission ability of *An. sinensis* has probably been underestimated in central China (Pan et al., 2012). In addition, the competence of *An. sinensis* (laboratory strain) to *P. vivax* was similar to *An. anthropophagus* when evaluated by a membrane feeding assay under experimental conditions (Zhu et al., 2013). The overall low vectorial capacity of *An. sinensis* may be related to tendencies for zoophilic feeding behavior in some study sites. Interestingly, the enhancement in vector capacity of *An. sinensis* was attributed to the local resident habits and the decline in the number of large livestock leading to the reduction of the traditional biological barriers (Pan et al., 2012). Vector capacity of *An. sinensis* can also depend on other factors, such as the larval environment (Moller-Jacobs et al., 2014).

Different *An. sinensis* populations in China exhibit variability in morphology, chromosomes, ecology, vector capacity, mitochondrial DNA, and even entire genomic DNA composition (Feng et al., 2017). Understanding the molecular and genetic mechanisms that determine variability in transmission efficiency and mosquito susceptibility could aid in the development of novel vector control strategies. A draft genome and transcriptome are currently available and many genes, including small RNA genes have also been identified. However, only 12 genes have been experimentally studied. This is a huge knowledge gap compared to the detailed analysis of variation in gene expression throughout development or related to pathogen infections which have been studied in other mosquito species. Approximately 25% of the genes showed a complex pattern of changes in gene expression during the different life stages of *An. gambiae* (Strode et al., 2006), and a total of 94 molecules pertaining to parasite infection have been validated (Sreenivasamurthy et al., 2013). Attention also should be focused on the new research directions such as gut-microbiome–parasite interactions, genetics of mosquito behavior, epigenetics and non-coding RNA. Identified molecules will provide useful tools for further functional analysis of the genetic, ecological and immune aspects. Determining the interactions and employing these as efficient resources for malaria intervention will require more research.

As China moves toward malaria elimination, it will be necessary to continuously update and summarize *An. sinensis* vector research. Climate and environmental landscapes continue to change and appropriate entomological surveillance and evaluation will alert researchers to biological and bionomic changes. The value of applying new tools generated from molecular studies of *An. sinensis* malaria transmission could be highly significant.

## Conclusion

This review provides current information on biology, bionomics, and molecules relevant to *An. sinensis* in China. Traditional research has provided a wealth of information on *An. sinensis* biology and bionomics. However, there is a lack of quantitative information required to characterize mechanisms of physiology and developmental biology and interactions with parasites. Future studies should fill these knowledge gaps. An integrated understanding of biology, bionomics, and molecules may yield more effective control strategies to facilitate malaria elimination in China.

## Author Contributions

XF, ShaZ, WH, and ShuZ were involved in the conception of the idea. XF and ShaZ conducted the literature searches. XF and WH participated in writing the final manuscript. ShuZ, FH, LZ, JF, HZ, and ZX reviewed and analyzed the documents. All authors read and approved the final manuscript.

## Conflict of Interest Statement

The authors declare that the research was conducted in the absence of any commercial or financial relationships that could be construed as a potential conflict of interest. The reviewer MDE-G and handling Editor declared their shared affiliation, and the handling Editor states that the process nevertheless met the standards of a fair and objective review.

## Footnotes

1 https://www.ncbi.nlm.nih.gov/assembly/GCA_000472065.2/

## Abbreviations

aa: amino acid
*Ae. Aegypti*: *Aedes aegypti*
*An. Gambiae*: *Anopheles gambia*
*An. Sinensis*: *Anopheles sinensis*
AQP: aquaporin
CCEs: carboxylesterases
cDNA: complementary DNA CPF
CPF: cuticular protein family
*Cu. Fatigans*: *Culex fatigans*
EIR: entomological inoculation rate
GFATM: The Global Fund to Fight AIDS, Tuberculosis and Malaria
GO: gene ontology
GSTs: glutathione-*S*-transferases
HSP40: heat shock protein 40
IRE-BP1: Iron responsive element binding protein 1
IRS: indoor residual spraying
ITS2: Internal transcribed spacer 2 capacity
JEV: Japanese encephalitis virus
KEGG: Kyoto Encyclopedia of Genes and Genomes
LLINs: long-lasting insecticidal nets
miRNAs: microRNAs
NGS: Next Generation Sequencing
nt: nucleotide
OBP: odorant-binding proteins
ORF: open reading frame
*P. f*: *Plasmodium falciparum*
*P. v*: *Plasmodium vivax*
PCR: polymerase chain reaction
sRNAs: small RNAs
SRPN14: serine protease inhibitor 14
TH: tyrosine hydroxylase
UTR: Untranslated Regions
VC: vector competence
WHO: World Health Organization
